# Ruptured middle cerebral artery aneurysms with a concomitant intraparenchymal hematoma: the role of hematoma volume

**DOI:** 10.1007/s00234-018-1978-4

**Published:** 2018-01-22

**Authors:** I. A. Zijlstra, W. E. van der Steen, D. Verbaan, C. B. Majoie, H. A. Marquering, B. A. Coert, W. P. Vandertop, R. van den Berg

**Affiliations:** 1 Department of Radiology, Academic Medical Center Amsterdam, Meibergdreef 9, 1105 AZ Amsterdam, The Netherlands; 2 Department of Neurology, Academic Medical Center, Amsterdam, The Netherlands; 3 Department of Neurosurgery, Academic Medical Center, Amsterdam, The Netherlands; 4 Department of Biomedical Engineering and Physics, Academic Medical Center, Amsterdam, The Netherlands

**Keywords:** Aneurysm, Hematoma, Middle cerebral artery, Ruptured, Subarachnoid hemorrhage

## Abstract

**Purpose:**

To study whether clinical outcome data from our patient cohort could give support to the new recommendation in the AHA/ASA guidelines for the management of aneurysmal subarachnoid hemorrhage that states “that microsurgical clipping may receive increased consideration in patients with ruptured middle cerebral artery (MCA) aneurysms and large (>50 mL) intraparenchymal hematomas”, while clinical outcome data supporting this recommendation are sparse.

**Methods:**

We reviewed the clinical and radiological data of 81 consecutive patients with MCA aneurysms and concomitant hematomas admitted between January 2006 and December 2015. The relation between (semi-automatically quantified) hematoma volume (< or > 50 ml), neurological condition on admission (poor: GCS < 8 or non-reactive pupils), treatment strategies (no treatment, coiling, or clipping with or without decompression and/or clot removal), and outcome (favorable: mRS score 0–3) was evaluated.

**Results:**

Clinical outcome data were available for 76 patients. A significant difference in favorable outcome (17 vs 68%) was seen when comparing patients with poor and good neurological condition on admission (*p* < 0.01). Patients with hematomas > 50 ml had similar outcomes for coiling and clipping, all underwent decompression. Patients with hematomas < 50 ml did not show differences in favorable outcome when comparing coiling and clipping with (33 and 31%) or without decompression (90 and 88%).

**Conclusion:**

Poor neurological condition on admission, and not large intraparenchymal hematoma volume, was associated with poor clinical outcome. Therefore, even in patients with large hematomas, the neurological condition on admission and the aneurysm configuration seem to be equally important factors to determine the most appropriate treatment strategy.

## Introduction

The prognosis of patients with ruptured middle cerebral artery (MCA) aneurysms is worse when the subarachnoid hemorrhage is complicated by an intraparenchymal hematoma, with a reported 6 months’ mortality of 13–56%, even after aggressive treatment with decompression, clot removal, and clipping of the aneurysm [1–3]. Studies on clot removal and decompression after coiling of ruptured aneurysms report somewhat more promising results, with a 6 months’ mortality of 20–30%, although these studies include aneurysms on all locations, including the MCA [4–6].

A new recommendation in the guidelines for the management of aneurysmal subarachnoid hemorrhage of the American Heart Association states that microsurgical clipping may receive increased consideration in patients presenting with large (> 50 ml) intraparenchymal hematomas and middle cerebral artery aneurysms [7]. However, clinical outcome data supporting this recommendation are sparse and the reference that was used in this guideline does not clearly define hematoma volumes [8]. Other studies on patients with ruptured MCA aneurysms and intraparenchymal hematoma mostly focus on a subgroup of patients, such as patients with WFNS grades IV and V on admission, or on one of the several treatment strategies (coiling or clipping with or without clot removal and/or clot removal), due to which important information could be missing [1–5, 9].

For all patients admitted to our hospital with a ruptured MCA aneurysm and a concomitant intraparenchymal hematoma, we retrospectively evaluated the association between intraparenchymal hematoma volume, neurological condition on admission, and any combination of treatment options (no treatment, coiling or clipping, decompression, and clot removal) and clinical outcome.

## Methods

### Study design

This research was exempt from review by the local institutional ethical committee. The clinical charts and imaging studies (CT, MR, DSA) were reviewed of all consecutive patients with a CTA- or DSA-proven ruptured MCA aneurysm and a concomitant intraparenchymal hematoma admitted between January 2006 and December 2015 to our hospital, which acts as a tertiary referral center for patients with a SAH.

Demographic data, time of initial hemorrhage and hospital admission, Glasgow Coma Scale (GCS) score and WFNS score on admission, neurological findings, rebleeding, and the presence of an intraparenchymal hematoma were collected [10, 11]. If a patient was transferred to our hospital under sedation, the last known GCS score was used. Poor neurological condition on admission was defined clinically as a GCS score < 8 and/or abnormal pupil reactions. In case the neurological condition changed during admission and before treatment, the last scores before treatment were used for calculations.

The treatment strategies were divided into three categories: no (endovascular or surgical) treatment, coiling, or clipping of the aneurysm. Coiling and clipping could be accompanied with decompression and clot removal. Decompression was defined as a hemicraniectomy on the side of the intraparenchymal hemorrhage without replacing the bone flap in the same procedure or as clot removal with or without replacing the bone flap in the same procedure. The decision to perform decompression with or without clot removal was at the neurosurgeons discretion. Clot removal was scored as having been performed in case it was specifically mentioned in the operative report. In patients treated with decompression and clipping of the aneurysm, we secondarily evaluated whether the aneurysm had been considered suitable for coiling or not. Indications for extraventricular drain (EVD) placement were hydrocephalus and comatose condition. The EVD strategy was 15–20 cm H2O above Monro in patients with untreated aneurysms and 5–10 cm H2O above Monro in patients with treated aneurysms. Mannitol was only given in case of intractable raised intracranial pressure.

The intraparenchymal hematoma volume (in ml) and the adjoining intra-Sylvian hematoma volume was delineated by an “automatic hematoma segmentation algorithm” and corrected in consensus by two observers (IJZ and WS) on the baseline non-contrast CT-scan (5 mm slices) using ITK Snap version 3.4.0 (http://sourceforge.net/projects/itk-snap) [12]. As it is very difficult to differentiate blood in the Sylvian fissure from true intra-Sylvian hematoma, we assessed the total hematoma volume. The calculated volume was then dichotomized into < or > 50 ml, conforming to the new AHA/ASA guidelines [7].

The baseline CT-scan was used to assess hematoma side, hematoma location, midline shift, and hydrocephalus. A temporal lobe hematoma was defined as a hematoma located in the temporal lobe with or without secondary extension to the frontal or parietal lobe. Rebleeding was defined as an additional bleeding from the causative aneurysm after the initial bleeding, determined by an increase of blood on a plain head CT-scan, or by outflow of fresh blood from an external ventricular drain. In case of rebleeding before treatment, the blood volumes after rebleeding were used for analyses. In those patients who required decompression, the time interval between hospital admission and the start of surgery and decompression was measured. Decompression within 6 h of hospital admission was defined as urgent. If coiling was performed within this interval and prior to decompression, the delay between the coiling and the start of the decompression was also evaluated.

Complications (aneurysm rupture, procedure-related thromboemboli, possible procedure-related ischemia, and delayed cerebral ischemia (DCI)) were recorded. Procedure-related thromboembolic/ischemic events were defined as a visible thromboembolus on angiography during coiling or as permanent clipping of a vessel during surgery, or as new hypodensities in the treated vascular territory on a CT within 48 h after an otherwise uncomplicated procedure. DCI was defined as any new focal neurological deficit (motor, sensory, or speech), or a decrease of two points or more on the GCS, that could not be attributed to other causes such as hydrocephalus, electrolyte or metabolic disturbances, rebleeding, or post-treatment complications or infections, and lasted for at least 1 h [13].

We evaluated the overall clinical outcome after 3 to 6 months using the modified Rankin scale (mRS) [14]. Favorable outcome was defined as a mRS score of 0–3 in concordance with recent publications on the treatment of MCA aneurysms with associated hematomas [1, 15].

### Statistical analysis

Continuous variables were presented as mean (SD) for normally distributed variables and as median (IQR) for non-normally distributed variables. Continuous variables were tested using the Shapiro-Wilk test (W > 0.9 is considered as a normally distributed variable). Categorical variables were presented as percentages. Categorical variables were tested using the Fisher’s exact test. Values of *p* < 0.05 were considered statistically significant.

## Results

### Baseline characteristics and treatment complications

Eighty-one consecutive patients with a ruptured MCA aneurysm were identified. Five patients were lost to follow-up after transfers to other hospitals (four to other countries). Baseline characteristics on admission and complications after treatment of the remaining 76 patients are presented in Table 1. No significant differences were found between coiling and clipping in the analysis of all the variables. An intraprocedural rupture occurred in six (8%) patients, five (83%) during clipping and one (17%) during coiling of the aneurysm. The thromboembolic/ischemic complications occurred in 17 patients (22%), in six (35%) patients during coiling of the aneurysm, and in 11 (65%) patients during or after clipping of the aneurysm. In four (67%) of six patients, Reopro (5 or 10 mg) was given during the coiling procedure. In four patients, a decompression was performed after coiling of the aneurysm, in all cases because of the ischemic complication. In four (36%) out of 11 patients treated with clipping, the ischemia could be directly related to the clipping procedure. In the other seven (64%) cases, new hypodensities were seen on the CT made < 48 h after an otherwise uncomplicated procedure. In nine (81%) out of 11 patients with ischemic complications, decompression was performed after clipping of the aneurysm, because of the intraprocedural complication or because of postprocedural neurological deterioration.

**Table 1:** Baseline characteristics and treatment complications in 76 patients with a ruptured MCA aneurysm and a concomitant intraparenchymal hematoma

Demographic/clinical*	No treatment *n* (%)	Coiling *n* (%)	Clipping *n* (%)
Patients	11 (14)	28 (37)	37 (49)
Age in years (SD)	68 (11)	56 (14)	55 (11)
Female gender	8 (73)	23 (82)	26 (70)
Poor neurological condition on admission	9 (82)**	10 (36)**	16 (43)**
WFNS			
1	0	4 (14)	5 (14)
2	2 (18)	1 (4)	2 (5)
3	0	5 (18)	4 (11)
4	2 (18)	14 (50)	15 (41)
5	7 (64)	4 (14)	11 (30)
Radiological*			
Left-side hematoma	10 (91)	14 (50)	11 (30)
Temporal lobe hematoma***	8 (73)	21 (75)	31 (84)
Mean hematoma volume ml (SD)	30 (26)	29 (20)	27 (21)
Mean large (> 50 ml) hematoma volume ml (SD)	65 (10)	58 (13)	68 (13)
Midline shift > 2 mm	11 (100)	23 (82)	35 (95)
Intraventricular hematoma	9 (82)	13 (46)	25 (68)
Hydrocephalus	4 (36)	8 (29)	13 (35)
Complications*			
Intraprocedural rupture	0	1 (4)	5 (14)
Thromboemboli/ischemia†	0	6 (21)	11 (30)
Delayed cerebral ischemia	1 (9)	7 (25)	10 (27)

*No statistical significant differences were found between all analyzed variables

**Data missing in one patient

***All other hematomas were located in the frontal lobe

†(possible) procedure-related ischemia

#### Treatment strategies and clinical outcome

The clinical outcome after 3–6 months’ follow-up in relation to treatment strategy is presented in Table 2. Of the 11 (14%) patients who received no treatment, 10 patients had absent brain stem reflexes and subsequently died in hospital. One patient was not treated because of old age (89 years); she was living independently (mRS score 2) in a nursery home 6 months after discharge.

**Table 2:** Clinical outcome after 3–6 months’ follow-up in relation to treatment strategy in 76 patients with a ruptured MCA aneurysm and an associated intraparenchymal hematoma

Treatment strategy	Total group *n* (%)	Clinical outcome *n* (%)		
		mRS 0–3	mRS 4–5	mRS 6
	76	32 (40)	15 (19)	29 (38)
No treatment	11 (14)	1 (9)	0	10 (91)
Coiling	28 (37)	15 (54)	8 (29)	5 (18)
No decompression	10 (13)	9 (90)*	1 (10)	0
Decompression	9 (12)	2 (22)*	4 (44)	3 (33)
+ clot removal	9 (12)	4 (44)	3 (33)	2 (22)
Decompression < 6 h	6 (8)	2 (33)	3 (50)	1 (17)
Clipping	37 (49)	16 (43)	7 (19)	14 (38)
No decompression	8 (11)	7 (88)*	0	1 (13)
Decompression	9 (12)	2 (22)*	2 (22)	5 (56)
+ clot removal	20 (26)	7 (35)	5 (25)	8 (40)
Decompression < 6 h	15 (20)	3 (20)	5 (33)	7 (47)

*mRS* modified Rankin scale

*Significant difference in favorable outcome between no decompression and decompression (*p* < 0.01)

Overall, there was no significant difference in favorable outcome or 6 months’ mortality between patients treated with coiling or clipping. Of the 65 treated patients, 19 (29%) died: five (18%) after coiling and 14 (38%) after clipping. Patients whose treatment included decompression showed a significantly worse favorable outcome compared to patients who were treated without decompression, and this was seen for both clipping and coiling (*p* < 0.01).

Urgent decompression was accomplished faster in a strict surgical approach. The mean (SD) (min-max) time interval between hospital admission and the start of the urgent surgical decompression was 161 (102) minutes (34–331). The mean (SD) (min-max) time interval between hospital admission and the start of the urgent surgical decompression after coiling was 268 (53) minutes (205–331).

#### Hematoma volume

Clinical outcome after 3–6 months’ follow-up in relation to treatment strategies and blood volumes < 50 or > 50 ml is presented in Table 3. Overall, no significant differences in favorable outcome were seen when comparing patients with a hematoma volume > 50 ml (29%) or < 50 ml (45%).

**Table 3:** Clinical outcome after 3–6 months’ follow-up in relation to treatment strategies and blood volumes > or < 50 ml in 76 patients with a ruptured MCA aneurysm and an associated intraparenchymal hematoma

	> 50 ml, *n* (%)				< 50 ml, *n* (%)			
	Total	Clinical outcome			Total	Clinical outcome		
		mRS 0–3	mRS 4–5	mRS 6		mRS 0–3	mRS 4–5	mRS 6
	14 (18)	4 (29)	3 (21)	7 (50)	62 (82)	28 (45)	12 (19)	22 (35)
No treatment	3(21)	0	0	3 (100)	8 (13)	1 (13)	0	7 (88)
Coiling	6 (43)	2 (33)	2 (33)	2 (33)	22 (35)	13 (59)	6 (27)	3 (14)
No decompression	0	0	0	0	10 (45)	9 (90)	1 (10)	0
Decompression*	6 (100)	2 (33)	2 (33)	2 (33)	12 (55)	4 (33)	5 (42)	3 (25)
Clipping	5 (36)	2 (40)	1 (20)	2 (40)	32 (52)	14 (44)	6 (19)	12 (38)
No decompression	0	0	0	0	8 (25)	7 (88)	0	1 (13)
Decompression*	5 (100)	2 (40)	1 (20)	2 (40)	24 (75)	7 (29)	6 (25)	11 (46)

No statistical significant differences were found between all analyzed variables

*mRS* modified Rankin scale

*With or without clot removal

All treated patients with a hematoma volume > 50 ml were treated with decompression (with or without clot removal). No significant difference in clinical outcome was seen between coiling and clipping (with or without decompression and/or clot removal) in patients with a hematoma volume of > 50 ml. Although the mortality rate in patients with a hematoma volume < 50 ml was higher after clipping (38%) than after coiling (14%), this difference was not significant.

Clinical DCI occurred in two (14%) patients with a hematoma volume of > 50 ml and in 16 (26%) patients with a hematoma volume of < 50 ml, this difference was not significant.

#### Neurological condition on admission

Data on pupil reactions on admission were missing in three patients with a GCS > 8. The results of the remaining 73 patients are presented in Table 4.

**Table 4:** Clinical outcome after 3–6 months’ follow-up in relation to treatment strategies and neurological condition before treatment in 73 patients with a ruptured MCA aneurysm and an intraparenchymal hematoma

	Poor neurological condition, *n* (%)				Good neurological condition, *n* (%)			
	Total	Clinical outcome			Total	Clinical outcome		
		mRS 0–3	mRS 4–5	mRS 6		mRS 0–3	mRS 4–5	mRS 6
	35 (48)	6 (17)**	8 (23)	21 (60)	38 (52)	26 (68)**	7 (18)	5 (13)
No treatment	9 (26)	0	0	9 (100)	1 (3)	1 (100)	0	0
Coiling	10 (29)	1 (10)	5 (50)	4 (40)	17 (45)	14 (82)	3 (18)	0
No decompression	0	0	0	0	10 (59)	9 (90)	1 (10)	0
Decompression^†^	10 (100)	1 (10)	5 (50)	4 (40)	7 (41)	5 (71)	2 (29)	0
Clipping	16 (46)	5 (31)	3 (19)	8 (50)	20 (53)	11 (55)	4 (20)	5 (25)
No decompression	2 (13)	2 (25)	0	0	6 (30)	5 (83)	0	1 (17)
Decompression^†^	14 (87)	3 (21)	3 (21)	8 (57)	14 (70)	6 (43)	4 (29)	4 (29)

Data on pupil reactions on admission missing in three patients

*mRS* modified Rankin scale

**Significant difference (*p* < 0.01), also when only treated patients are analyzed (*p* < 0.01)

^†^With or without clot removal

A significant difference in favorable outcome was seen when comparing patients with poor (17%) and good (68%) neurological condition on admission (*p* < 0.01). The difference (24 vs 66%) remained significant (*p* < 0.01) when only treated patients were analyzed.

Clinical DCI occurred in five (14%) patients with poor neurological condition and in 11 (29%) patients with good neurological condition on admission, this difference was not significant.

No significant difference in favorable outcome was seen in patients with poor or good neurological condition on admission who were treated with coiling or clipping, with or without decompression. There also was no significant difference in mortality rate in these patients.

All five patients with good neurological condition on admission who died after clipping of the aneurysm suffered from DCI.

#### Hematoma volume and neurological condition on admission

**Fig. 1:**
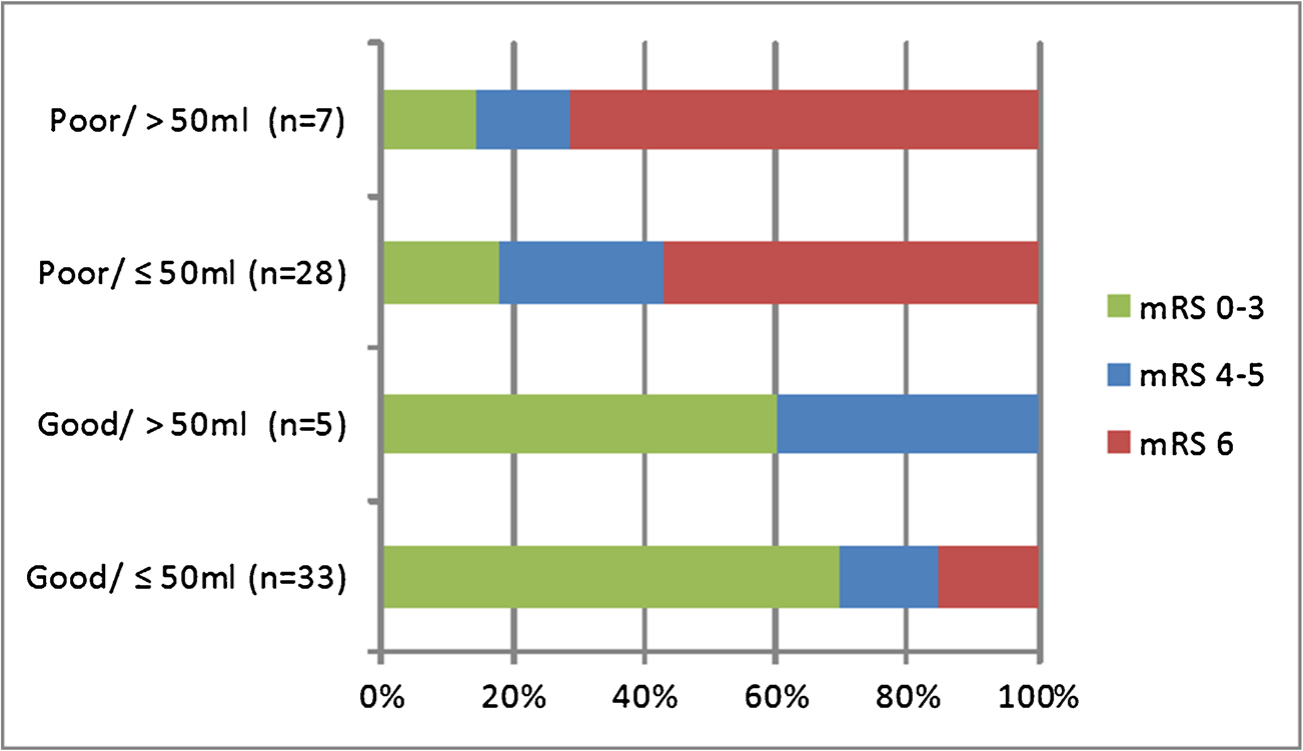
Neurological condition and hematoma volume in relation to clinical outcome after 3–6 months’ follow-up in 73* patients with a ruptured MCA aneurysm and an associated intraparenchymal hematoma. *Data missing in three patients. No significant differences were found in the analysis of all the variables. *mRS* modified Rankin Scale

When the combination of neurological condition and intraparenchymal hematoma volume on admission were related to clinical outcome, neurological condition on admission (poor vs good) was more discriminative in determining clinical outcome than intraparenchymal hematoma volume (< or > 50 ml) (Fig. 1). In the good grade patients with small hematomas, 16 (48%) out of 33 patients were treated with decompression, four (25%) after coiling and 12 (75%) after clipping. In ten (30%) out of these 33 patients, a hemicraniectomy without replacing the bone flap was performed, in seven (70%) patients after clipping and in three (30%) after coiling, all due to complications/neurological deterioration during or after the procedure. In the other six patients, clot removal was performed during or after the aneurysm treatment. Favorable outcome was reached in 12 (86%) out of 14 patients after coiling, in 10 (56%) out of 18 patients after clipping, and in one (100%) untreated patient. The difference between coiling and clipping was not significant. Three out of five (60%) patients with a hematoma volume > 50 ml and a good neurological condition on admission had favorable clinical outcome, two after coiling and one after clipping with decompression and clot removal.

## Discussion

In patients with a ruptured MCA aneurysm and a concomitant intraparenchymal hematoma, neurological condition on admission was strongly related with clinical outcome, whereas hematoma volume was not. Moreover, many patients with a poor neurological condition on admission had a hematoma volume < 50 ml. No significant difference in clinical outcome between coiling and clipping was found in patients with hematoma volumes of > or < 50 ml.

The 2012 American Heart Association guidelines for the management of aneurysmal subarachnoid hemorrhage states that microsurgical clipping may receive increased consideration in patients presenting with large (> 50 mL) intraparenchymal hematomas and middle cerebral artery aneurysms [7]. However, the reference that was used in this guideline does not clearly define hematoma volumes [8]. In our study, we observed that all patients with a hematoma volume of > 50 ml were treated with decompression, irrespective from neurological condition on admission. Simultaneously, all but two patients with poor neurological condition on admission were treated with decompression. While the number of patients presenting with a hematoma volume > 50 ml was quite low in comparison with those presenting in a poor neurological condition, this limits the numerical importance of volume, as compared to neurological condition to determine the need for decompressive surgery. Clinical outcome was worse after decompression also in patients with hematomas < 50 ml, who were in good neurological condition before treatment, because in these patients, decompression was often performed after complications or neurological deterioration during or after the coiling or clipping procedure. The higher complication rate after clipping might be explained by the coil-first policy in our hospital leaving the most challenging aneurysms for surgical treatment. Furthermore, a considerable proportion (4/11) of patients underwent an emergency operation for impending cerebral herniation, precluding any coiling procedure. Additionally, it is difficult to differentiate between edema, normal parenchymal changes after hematoma removal, and infarcts on a post procedural CT. Therefore, we might have overrated the clipping related ischemia. We suggest to perform a perfusion CT after these procedures to overcome this problem in future research. The higher hematoma decompression rate during clipping can be explained by the fact that the surgeon almost automatically comes across the hematoma when opening the dura.

Decompression can be preceded by coiling of the aneurysm, but this strategy is strongly dependent on the aneurysm configuration. The rationale of coiling first, despite the delay to decompression, is to secure the aneurysm prior to decompressive surgery, with or without hematoma evacuation, diminishing the risk of intraoperative rupture of the aneurysm. With respect to coiling and clot removal in patients with ruptured aneurysms, there are studies reporting 25–60% favorable outcome rates after coiling, but these included aneurysms in all locations. There are no studies solely reporting on this treatment strategy in patients with ruptured MCA aneurysms [4–6].

When coiling is not possible, or when, based on neurological condition on admission, fast decompressive surgery is considered more important than primarily securing of the aneurysm, surgery is the preferred initial treatment. When we compare our outcome results with published data on surgical treatment of ruptured MCA aneurysms with a concomitant intraparenchymal hematoma (clipping in any combination with decompression and clot removal), one large study (144 patients) reported a higher overall mortality of 49% [2]. Several smaller studies reported lower mortality rates (13–29%) with favorable outcome rates ranging from 26 to 54% [3, 6, 9]. Patient selection may explain these differences. One study, in Hunt & Hess III-V patients and a hematoma volume > 30 ml, reported a mortality rate of 25–30%, but also included aneurysms on other locations [6]. In another study, of 24 Hunt & Hess grade II-V patients, a similar (29%) mortality rate was found [9]. None of these studies divided the hematomas in >/< 50 ml.

We chose mRS 0–3 as favorable outcome according to recent literature on treatment of ruptured MCA aneurysms with a concomitant hematoma [1, 15]. An additional argument is that the vast majority of the patients with a ruptured MCA aneurysm and a concomitant hematoma are admitted with a poor WFNS score of four or five, in our study 70% of the patients. The fact that patients are in a poor clinical condition on admission after a serious and life-threatening event, automatically leads to low expectations for a good outcome, not only with the doctors, but especially so with the patient and his or her family.

A limitation of our study is its retrospective nature, leading to missing data. Due to missing data the time from SAH ictus to decompression could not be analyzed, nor the occurrence of seizures. As of January 2011, a prospective database is maintained to minimize such data drop-outs. Furthermore, the sample size of our study is small, and therefore, the results have to be interpreted with some caution. The number of patients was not sufficient to perform a multivariate statistical analysis to detect possible confounding factors and to evaluate the association between both neurological status on admission and hematoma volume with clinical outcome due to which we had to analyze these factors as independent factors in a group comparison. Strength of our study is that we describe a consecutive patient cohort that was admitted over a period of 10 years’ time in a tertiary center using a multidisciplinary approach for each individual patient. Also there are very little other studies describing such a cohort of patients with ruptured middle cerebral artery aneurysms and a concomitant hematoma. Another strength is the very precise hematoma volume measurement. The hematoma volumes in our study are much lower than those reported elsewhere [1, 16]. For instance, the mean (SD) hematoma volume in the study by Stapleton et al. was 100 + 77 ml, while in our study, the mean (SD) hematoma volume (> 50 ml) was 68 + 15 ml and no patient had a hematoma volume of > 100 ml [15]. The difference can be explained by the method of volume measurement. In most studies, this is done with the ABC/2 method, or the pi4rABC method. Both are known to overrate the hematoma volume and small errors in measurement can lead to a high variation in volume [17]. Even though hematoma volume has no strong relation with outcome, we do propose a more standardized way to measure the hematoma volume to allow for better comparison of future studies. One such method to assess the total volume of subarachnoid hemorrhage has recently been published and shows a high degree of reproducibility [12]. This might lead to an improved prediction model, in which the total amount of blood in the different compartments (subarachnoid, parenchymal, and intraventricular) can be added. The clinical relevance is supported by a study reporting that Sylvian hematomas without an intraparenchymal component can predict a favorable outcome in poor-grade aneurysmal SAH patients [15]. The high mortality rate in our study in clipped patients with a GCS > 8 and a hematoma volume < 50 ml was related to the occurrence of DCI and might be related to the distribution of blood between the subarachnoid and parenchymal compartments. This is in accordance with data from an earlier study in which a negative association of the existence of an intraparenchymal hematoma was seen in relation to the occurrence of clinical DCI [18]. The absence of clinical DCI in the patients with poor neurological condition on admission and a hematoma volume > 50 ml group can be explained by the poor outcome rate, as most patients either died within 3 days, or were in a too bad condition to discover clinical signs of DCI.

According to our results, neurological condition on admission together with the aneurysm configuration seems to be more important than hematoma volume to determine the best possible treatment strategy. The problem to study this specific patient group with ruptured aneurysms with concomitant hematomas is the large diversity in approach and experience which severely limits the design of a multicenter prospective trial. We therefore are limited to observational studies. However, we can improve on these studies by standardized registration of patients in prospective databases.

## Summary

In patients with a ruptured middle cerebral artery aneurysm and a concomitant intraparenchymal hematoma, neurological condition on admission, and not large hematoma volume was associated with poor clinical outcome. The decision to perform decompressive surgery should be based more on neurological condition than on hematoma volume, especially after coiling of the aneurysm. Whether the decompression is combined with coiling or clipping of the aneurysm can be decided by the local neurovascular team based on the aneurysm configuration and the local expertise.
